# Methods of Isolation, Characterization and Expansion of Human Adipose-Derived Stem Cells (ASCs): An Overview

**DOI:** 10.3390/ijms19071897

**Published:** 2018-06-28

**Authors:** Paola Palumbo, Francesca Lombardi, Giuseppe Siragusa, Maria Grazia Cifone, Benedetta Cinque, Maurizio Giuliani

**Affiliations:** 1 Department of Life, Health & Environmental Sciences, University of L’Aquila, Building Delta 6, Coppito, 67100 L’Aquila, Italy; paola.palumbo@univaq.it (P.P.); francesca.lombardi@univaq.it (F.L.); giuseppe.siragusa@graduate.univaq.it (G.S.); mariagrazia.cifone@univaq.it (M.G.C.); benedetta.cinque@univaq.it (B.C.); 2 Unit of Plastic and reconstructive surgery, Casa di Cura “Di Lorenzo” SrL, Via Vittorio Veneto 37, Avezzano, 67051 L’Aquila, Italy

**Keywords:** human adipose-derived stem cells, harvesting, isolation, characterization, expansion

## Abstract

Considering the increasing interest in adipose-derived stem cells (ASCs) in regenerative medicine, optimization of methods aimed at isolation, characterization, expansion and evaluation of differentiation potential is critical to ensure (*a*) the quality of stem cells also in terms of genetic stability; (*b*) the reproducibility of beneficial effects; and (*c*) the safety of their use. Numerous studies have been conducted to understand the mechanisms that regulate ASC proliferation, growth and differentiation, however standard protocols about harvesting and processing techniques are not yet defined. It is also important to note that some steps in the procedures of harvesting and/or processing have been reported to affect recovery and/or the physiology of ASCs. Even considering the great opportunity that the ASCs provide for the identification of novel molecular targets for new or old drugs, the definition of homogeneous preparation methods that ensure adequate quality assurance and control, in accordance with current GMPs (good manufacturing practices), is required. Here, we summarize the literature reports to provide a detailed overview of the methodological issues underlying human ASCs isolation, processing, characterization, expansion, differentiation techniques, recalling at the same time their basilar principles, advantages and limits, in particular focusing on how these procedures could affect the ASC quality, functionality and plasticity.

## 1. Adipose-Derived Stem Cells (ASCs)

Adipose-derived stem cells (ASCs) are adult multipotent cells with homing, immunomodulation, promotion of tissue repair and regeneration properties. Moreover, their clinical use, unlike embryonic stem cells, is less associated with ethical controversies being harvested from autologous adult fat. ASC therapeutic potential is derived from their natural ability to maintain homeostasis being able to migrate to an area of injury then facilitating tissue repair, also exerting immunomodulatory effects. Allogeneic ASCs also carry a minimal rejection risk because they lack the ability to express MHC-II and express low levels of MHC-I proteins [1,2]. Adipose tissue is an excellent source of autologous mesenchymal stem cells (MSCs) which can be harvested easily compared with bone marrow-derived stem cells with a lower risk of complications for the patient. The International Federation for Adipose Therapeutics and Science (IFATS) and the International Society for Cellular Therapy (ISCT) proposed three minimal criteria for the definition of ASCs: (1) plastic adherence; (2) expression of CD73, CD90, and CD105, and lack of expression of CD11b, CD14, CD19, CD45, and HLA-DR; and (3) differentiation potential into preadipocytes, chondrocytes, and osteoblasts [1,2]. In this review, the main literature reports are collected to provide a detailed overview of the methodological issues underlying human ASCs isolation, processing, characterization, expansion, and differentiation techniques.

## 2. Sources of ASCs and Isolation Procedures

The viability, yield, proliferative index and stemness of ASCs can be influenced by the type of harvesting procedure. ASCs can be isolated from adipose tissue through previous surgical resection or liposuction. The latter procedure is mainly preferred being a safe, well-tolerated, slightly invasive procedure able to allow a high yield of stromal/stem cells. In a recent article, Bajek et al. [3] have comparatively evaluated the biological properties of ASCs in terms of clonogenicity, proliferation rate, doubling time, multilineage differentiation, and senescence potential after harvesting through the following three approaches: surgical resection, power-assisted liposuction (PAL), and laser-assisted liposuction (LAL). The authors show evidence that the method of ASC collection can affect the number of isolated cells, clonogenicity, and doubling time, concluding that, at the current state of knowledge, the best method of ASC collection for clinical purposes is PAL due to high proliferation potential and slow senescence of isolated cells.

**Figure 1:**
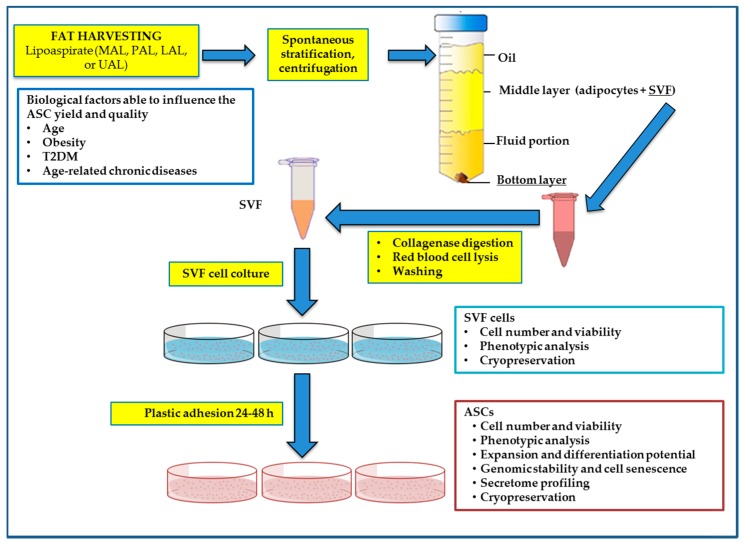
Schematic diagram of the workflow for stromal vascular fraction (SVF) and adipose-derived stem cell (ASC) isolation from lipoaspirate samples.

Several approaches for ASC isolation have been reported [4,5] but data comparing the efficacy of various methods are still not available; therefore, no standardized method exists. The protocol described in 2001 by Zuk et al., is still considered the most widely used method for ASC isolation [6]. The method proposed by Zuk et al., provides that lipoaspirate samples are extensively washed with equal volumes of phosphate-buffered saline (PBS), and then digested at 37 °C for 30 min with 0.075% collagenase, able to degrade the tight junctions and the components of the extracellular matrix. When enzymatically digested, lipoaspirate yields a heterogeneous population of many cell types (preadipocytes, fibroblasts, vascular smooth muscle cells, endothelial cells, resident monocytes/macrophages, lymphocytes, and ASCs), known as stromal vascular fraction (SVF). Enzyme activity is then neutralized with addition of Dulbecco’s modified Eagle’s medium (DMEM) containing 10% FBS and the cell suspension centrifuged at 1200× *g* for 10 min to obtain a high-density SVF pellet. The pellet is then suspended in NH_4_Cl and incubated at room temperature for 10 min to lyse contaminating red blood cells. The SVF is collected by centrifugation, filtered through a 100-μm nylon mesh to remove undigested tissue fragment and incubated overnight at 37 °C and 5% CO_2_ in control medium (DMEM, 10% FBS, 1% antibiotic/antimycotic solution). The adherent cells are kept in standard culture conditions until they reached sub-confluence (80–90%). Regarding the step related to erythrocyte removal, Li et al. [7] have recently compared two methods for red blood cell lysis with NH_4_Cl or NaCl hypotonic solution, evaluating their effects on lipoaspirate-derived ASC functionality. The authors conclude that hypotonic NaCl solution is more effective in ASC purification and meets the clinical safety standards of good manufacturing practice/good clinical practice guidelines. SVF can be extracted from lipoaspirates after density-gradient centrifugation, spontaneous stratification or filtration and cell washing. With reference to the step of density-gradient centrifugation or spontaneous stratification, in a previous report [8], our group has analyzed the influence of different handling methods on SVF cell and adipocyte yield and viability. In particular, two common fat processing techniques, i.e., lipoaspirate spontaneous stratification at different times and centrifugation at several speed forces, have been analyzed on (a) adipocytes and SVF cell number; (b) isolated ASC number; (c) ASC plastic adhesion ability; and (d) ASC differentiation potential. The spontaneous stratification procedure at 10, 20, or 30 min has been compared to centrifugation at different speeds (90, 400, or 1500× *g*). Results show that spontaneous stratification, at 20 or 30 min carries to a yield of ASCs comparable with that obtained after centrifugation at 90 or 400× *g* for 3 min. The ASCs successfully differentiate into adipogenic, osteogenic, and chondrogenic lineages, suggesting that the ability to differentiate is not influenced by any of the handling methods. Taken together, our results also suggest that the middle layer obtained from lipoaspirate samples after either spontaneous stratification at 20 min or centrifugation at 400× *g*, is enough to provide a good amount of ASCs and to preserve the adipocyte integrity, showing that both approaches are effective (Figure 1). Clearly, the technique of centrifugation can surely allow the surgical team a significant time saving for fat grafting, when the appropriate equipment is available.

Since Zuk et al. [6,9] many modified experimental procedures have been proposed [10]. However, the enzymatic digestion-based methods are mainly intended for experimental purposes, and are considered less suitable in clinical practice. The clinical use of proteolytic enzymes, e.g., trypsin-EDTA solution, dispase or collagenase, being able to negatively affect cell viability and surface antigens, is indeed considered critical by some Authors [11]. However, several clinical studies that used enzyme digested SVF have been published [12,13,14] and the results described below.

The isolation of ASCs using non-enzymatic methods have been described by several groups [15,16,17,18,19]. ASCs present in the infranatant layer of the suction canister after liposuction can be expanded ex vivo and show a phenotypic and differentiation potential similar to ASCs isolated via collagenase digestion, but their number appears to be significantly lower [6]. Busser et al. [20] have proposed a new one-step and quickly performed method to isolate ASCs from lipoaspirate without the collagenase digestion step comparing the collagenase methods with explant culture protocol. Collagenase treatment affects the quantity and quality of isolated ASCs; besides, cells obtained without enzymatic treatment show a higher potential to support hematopoiesis and a lower percentage of CD34 cells, thus supporting the quality, goodness, and effectiveness of the explant culture method.

Currently, several standardized protocols for fat grafting are available aimed at not only soft tissue augmentation but also tissue regeneration, thus promoting considerable interest in basic sciences and clinical research. The regenerative properties are closely linked to heterogeneous SVF cell population of grafted lipoaspirate, extracellular matrix and soluble mediators. The critical element in fat grafting consists on the rate of fat engraftment depending also on ASC richness [21]. Most surgical procedures for fat grafting are based on several easy approaches, such as simple lipoaspirate, gravity separation, filtration or centrifugation, all aiming to remove undesired components and to obtain fat tissue condensation with a better concentration of SVF, as source of ASC. A new procedure called cell-assisted lipotransfer (CAL) has been proposed to enrich the aspirate fat with SVF obtained after enzymatic isolation, improving the long-term volume retention [12,13]. To date, clinical devices for SVF isolating and obtaining enriched fat grafts in stem cells are available with different performance primarily in terms of cell yield, effectiveness and safety. In a study of Domenis et al. [14], the classical Coleman’s procedure [22] is compared to three devices for clinic use examining stem cell yield, proliferation and differentiation potential. Two devices perform an enzymatic isolation of SVF cells, while the third consists in a filtration system able to obtain mechanically the SVF cells. The SVFs obtained from these three systems are added to a remaining part of lipoaspirate, then the authors compared for each device yield, immunophenotype and colony-forming assay of SVF in standard lipoaspirate and SVF-enriched lipoaspirate. The results show that the lipoaspirates enriched with enzymatic methods have a significantly higher number of cells with respect to non-enriched lipoaspirate or enriched with a mechanical procedure, while no difference has been reported in stemness marker expression. Besides, the cells isolated from the lipoaspirates enriched with enzymatic methods, show a significant colony-forming efficiency together with high levels of Nanog, SOX2 and Oct4 after 1 week of seeding. In the same study, the authors have examined phenotype and function of ASCs isolated from non- and enriched lipoaspirate, at the third passage of culture showing that ASCs from enriched enzymatically lipoaspirate maintain higher Nanog and Oct4 expression levels. Functionally, the ASCs extracted from enriched lipoaspirate duplicate faster and are characterized by a higher ability to differentiate with respect to non-enriched lipoaspirate or mechanical enrichment procedures. Nevertheless, according to the regulations set forth by the US Food and Drug Administration (FDA), the fat graft must be minimally manipulated, enzyme-free and used in the same surgical procedure, therefore in clinical practice non-enzymatic methods are used. Recently innovative technology based on mechanical disruption of adipose tissue in closed systems has been reported [23]. In a recent study, the advantages derived by a simple device able to fat harvest with minimal manipulation through soft mechanical action, are described [24]. In particular, with this technology, cell components are preserved in the SVF, the enzyme absence maintains unaltered the exosome content, and the micro-fragmented tissue promotes the healing process of receiving tissue. Nonetheless, the same authors suggest better evaluating the performance of this device in multicenter studies. Although responding to clinical requirements, the non-enzymatic methods require large amounts of lipoaspirate and have lower efficiency in cell recovery when compared to enzymatic methods generally used in a research context [25].

## 3. Culture and Expansion of ASCs

ASCs are obtained by plating the SVF cells and can be either freshly isolated or cultured. Freshly isolated ASCs have been reported to appear more heterogeneous compared with the quite homogeneous cells harvested from cultured ASCs [1]. The SVF/ASCs culture protocol varies between laboratories and, currently, there is not a single available method. The most commonly used is a monolayer culture in standard medium supplemented with 10% fetal bovine serum [8,26]. In fact, regardless to isolation methods (enzymatic or non-enzymatic), SVF cells are seeded onto culture flasks and kept in sterile conditions at 37 °C, 5% CO_2_ in standard medium (i.e., DMEM or α-MEM) containing 10% FBS and 1% antibiotics to select adherent cells [8,27]. The remaining floating cells are removed 24–48 h later, and the flask is washed with medium to remove any debris. ASCs are maintained in medium supplemented with 10% FBS humidified at 5% CO_2_ 37 °C until sub-confluence at 80–90%, changing the medium every 2–3 days.

When sub-confluency is reached, adherent cells are harvested after enzymatic detachment, washed in PBS, counted and analyzed for their growth, clonogenicity, and antigen expression. Cells are expanded until passage 3 (P3) by replating at low density (~1000–4000 cells/cm^2^) [20,27]. Although the ASC isolation and expansion seem quite simple, their short life span limits not only their investigation in preclinical studies but also an exhaustive evaluation of their therapeutic potential. Indeed, like all adult cell types, ASCs also significantly decrease cell growth after a limited number of in vitro passages due to a cellular process termed replicative senescence. Nowadays, standard protocols for ASC seeding are lacking, as evidenced by the wide range of cellular concentrations (cell density/cm^2^) used for replating [20,27,28,29].

Also, considering the use of ASCs in regenerative medicine, an improvement in methods to assess their reproducibility, safety and quality of in vitro expanded cells is crucial. So, producing cells that are genetically stable is a step towards getting insurance that the cells would not transform, leading to genetically aberrated progeny when transplanted into the recipient. The production of clinical-grade ASCs in agreement with GMP procedures requires the careful identification and control of all the phases of cell manipulation. In addition to sterility tests, the methods of expansion must guarantee and maintain the phenotypic and functional characteristics of the cells as well as their genomic stability during the in vitro culture period. Few studies have focused on the safety of cultured human ASCs by molecular evaluation of genomic stability [30,31]. Due to the lack of international indications about genetic stability assessments, the karyotype technique is the most frequently used analysis [27]. Other useful methods are MicroSatellite Instability (MSI) analysis, telomere length and telomerase activity. Neri et al., using all the above mentioned methods, have evaluated at 14 day culture (end of P1) the potential susceptibility of in vitro expanded ASCs to genetic alterations [31]. No karyotype alterations, no change in telomere length and telomerase activity, no cases of MSI, no significant modification of mismatch repair gene expression, thus supporting the absence of genetic damage accumulation at the first culture passages, are detected. In a recent report, the ASC karyotype after expansion is analyzed between P12 and P14 in the presence of 5% supernatant rich in growth factors from platelets or 10% FBS. ASCs expanded in both media show unaltered phenotype as assayed by flow cytometry and no genetic lesion is observed [27]. Li et al. [30] have performed an explant method to isolate and expand in a serum-free medium ASCs according to current GMP guidelines. The biological characteristics, such as cell morphology, karyotype, cell cycle, immunophenotype and growth factors, are assessed at different culture passages (n. 1, 3, 5, 10, 15, 20). Even if the ASC proliferation rate begins to slow down significantly after 15 passages, no evident chromosomal aberrations are observed until 20 passages; also the gene expression level of p53, CCNE, Nanog and TERT basically remains stable at all passages, thus supporting the suggestion that ASCs manufactured through this protocol are safe and suitable for clinical transplantation.

ASCs are considered a promising tool for regenerative medicine mainly for their ability to secrete a wide range of bioactive factors as well as extracellular vesicles, able to affect both local and systemic physiological processes [32]. Indeed, scientific and clinical interest in the transplant of ASC-derived secretome or purified exosomes as a promising therapeutic strategy for the treatment of various diseases is increasing. The secretome is a complex set of exosomes and microvesicles secreted by living cells and can be isolated from all body fluids, and they carry a complex cargo mainly composed of biologically active proteins, lipids, nucleic acids [33,34]. Nowadays, a standardized handling method for ensuring a good quantity of ASC-derived secretome products for therapeutic approaches has not yet been established. Compared to cells, exosomes are more stable and easily storable, have lower maintenance cost and lower possibility of immune rejection following in vivo transplantation, and the molecules contained within the exosomes are better protected from degradation [32]. Several articles are focused on the characterization of ASC secretomic profile by proteomic approaches [35,36,37], but how the ASC isolation and expansion techniques could affect the secretome composition is not fully addressed.

## 4. Analysis of Phenotypic Profile of Stromal Vascular Fraction (SVF)/ASCs

The wide variety of methods of SVF/ASC harvesting and isolation had initially highlighted several issues in comparing study outcomes. Among the recommended ISCTS and IFAT criteria to define human SVF/ASCs, have included the negative or positive expression of distinct surface molecules. [1]. SVF cells are identified phenotypically as CD45^−^ CD235a^−^ CD31^−^CD34^+^. Added value may be provided by the following surface antigens: CD13, CD73, CD90 and CD105. In culture, ASCs retain markers in common with other mesenchymal stromal/stem cells (MSCs), including CD90, CD73, CD105, and CD44 and remain negative for CD45 and CD31. They can be distinguished from bone-marrow-derived MSCs by their positivity for CD36 and negativity for CD106.

The marker expression can be affected by in vitro culture and passage numbers [38]. Phenotype analysis is always performed in ASC studies just for confirming the mesenchymal nature of isolated cells. Few studies evaluate whether the tissue harvesting method can affect the phenotype. Bajek et al. [39] have compared the surface markers in ASCs obtained by mechanical or ultrasound-assisted liposuction (MAL, UAL) and selected at the second passage. 242 different markers using the BD LyoplateTM Human Cell Surface Marker Screening Panel. The expression of selected markers for MSC phenotype i.e., CD13, CD29, CD73, CD90, CD105 is similar in both ASC populations, nonetheless CD166 level is higher in UAL-derived ASCs. On the other hand, uncharacteristic markers such as CD31, CD45 and HLA-DR in UAL-derived ASCs are significantly higher than MAL-derived ASC population. However, the authors conclude that even if statistically significant differences are observed in 58 markers, the differences in the most important markers are rather small, thus concluding that the collection method does not significantly affect the ASC phenotypic profile. Bajek et al., have recently confirmed that different collection methods such as surgical resection, PAL, and LAL, do not alter the expression of major mesenchymal markers such as CD90 and CD44 on ASC population during long-term culture in vitro [3].

## 5. ASC Differentiation Potential

According to the criteria defined by IFATS/ISCT, to complete the ASC identification their multipotency and ability to differentiate to osteoblastic, chondrocytic and adipocytic lineages has to be assessed using standard in vitro tissue culture-differentiating conditions [40]. Currently, suitable commercial kits are proposed containing specially formulated media supplements and cytochemical staining solutions and/or a panel of antibodies for evaluating the differentiation that occurs in the different cell lineages. However, to precisely analyze the differentiation, a quantitative assessment is advised using lineage-specific gene or protein markers as proposed [1].

The osteogenic differentiation medium must contain: β-glycerophosphate, dexamethasone, ascorbic acid-2-phosphate and combinations of transforming growth factor-beta (TGF-β), bone morphogenetic proteins and vitamin D3 [1,40]. Recently, melatonin or a mixture of hyaluronic, butyric, and retinoic acids have been reported to efficiently promote an osteogenic patterning in human dental pulp stem cells by inducing the transcription of a gene program of osteogenesis [41,42]. The differentiation of ASCs into osteocytes is controlled by various transcription factors as such as Runt-related transcription factor 2 (Runx2), osterix, and β-catenin. The ASCs are incubated with osteogenic differentiation media for 14–21 days and then at 14 and 21 days of culture, the differentiation is firstly evaluated by staining with alizarin red or von Kossa. To confirm osteoblast phenotype, histochemistry or RT-PCR analysis are performed to evaluate specific gene and protein expression, such as key osteogenic transcription factor Runx2, alkaline phosphatase, bone sialoprotein, osteocalcin, osterix [1].

The adipogenic differentiation medium must be supplemented with 3-isobutyl-1-methyl-xanthine, insulin, indomethacin, triiodothyonine, Asc-2-P, basic fibroblast-growth factor (FGF)-basic, and the glucocorticoid dexamethasone [40]. The adipogenic differentiation is morphologically characterized by spherical shape with accumulating lipid droplets [8]. The transcription factor mostly implicated in the adipogenic differentiation results in the peroxisome proliferation-activated receptor γ (PPARγ) that is able to regulate expression of genes promoting the adipogenic process [1,40]. Adipogenic differentiation medium is used for culturing the stem cells for 7–21 days. The analysis of adipogenic phenotype is verified with oil-red or Nile red staining and then several biochemical markers are detected such as adiponectin, fatty acid binding protein 4, leptin, PPARγ, glycerol 3 phosphate dehydrogenase by histochemistry or reverse transcription polymerase chain reaction.

The chondrogenic differentiation medium must be completed by addition of ascorbic acid phosphate, dexamethasone, bovine serum albumin, linoleic acid, sodium pyruvate, transferrin, selenious acid, and TGF-β1 [40,43]. The SRY-related high mobility group-box gene 9 (*Sox9*) is one of the major transcription factors that regulates the chondrogenic commitment. The differentiation begins to appear when the cells change from a fibroblast-like morphology into a round shape. For chondrocyte differentiation the stem cells are incubated for 14–21 days. The chondrogenic phenotype is verified with alcian blue or Safranin O staining. In addition the aggrecan, collagen type II, *Sox9* markers are also evaluated [1]. 

The adipose stem cells have also the potential to differentiate into non mesodermal lineages including cardiomyocytes [44,45], smooth muscle cells [9,46], endothelial cells [45], and neurons [47]. Adipose tissue-derived MSCs can be differentiated into hepatocytes by using a cocktail of hepatocyte induction factors which comprises FGF1, FGF4 and HGF. This commitment is induced by factors like oncostatin M and dexamethasone [48]. Moreover, ASCs also have the properties to differentiate into endothelial cells and macrophages [49,50]. In addition, Bellei et al. [25] have reported that ASCs can differentiate into melanocytes in the presence of selected growth factors. Therefore, non-mesodermal and mesodermal lineage differentiation properties will likely be a central part of the next generation of ASC-based therapy.

## 6. SVF/ASC Cryopreservation

Basic and clinical research investigating SVF/ASCs is primarily performed on fresh specimens obtained principally by whole adipose tissue or lipoaspirate, but it is not always possible to work on fresh material. Often their use is indeed shifted temporally with respect to the time of collection. There are many reasons supporting the need to use frozen tissues or cells as an alternative approach and the possibility of storing purified SVF as well as ASCs by cryopreservation and freezing is another crucial step to optimize the study design, in particular for clinical applications.

The procedures for cryopreservation must be efficient and valid. For this reason it is central to evaluate several parameters including cell recovery, viability, phenotype, proliferation and multi-lineage differentiation potential. To date, only a few studies have investigated the effect of cryopreservation procedures on human ASCs. Devitt et al. [28] have investigated the effect of cryopreservation (−70 °C) of human whole adipose tissue on ASC isolation, viability and growth. The increase of cryopreservation time (range 2–1159 days), also inducing a progressive reduction of live ASC number and cell viability statistically significant just at a >2 year freezing period, does not affect either cell growth or stemness potential.

Recently, Zanata et al. [51] have investigated the effect of cryopreservation (4–6 weeks) on SVF viability and number, colony-forming unit ability and immunophenotypic profile. Three different conditions are compared: SVF from fresh lipoaspirate, SVF from cryopreserved lipoaspirate (−80° in 70% FCS, 20% DMSO, 10% stromal medium) and cryopreserved SVF (−80 °C in 80% FCS, 10% DMSO, 10% stromal medium) from fresh lipoaspirate. The lipoaspirate cryopreservation, while not affecting SVF cell yield when compared to fresh lipoaspirate-derived SVF, leads to a significant reduction of cell viability. Moreover, the colony-forming-unit percentage appears strongly reduced (~16 fold) in cryopreserved lipoaspirate-derived SVF. In terms of SVF cell surface markers, no relevant differences are shown with the exception of CD73 which was significantly upregulated in cryopreserved lipoaspirate-derived SVF. The cryopreservation of SVF does not influence cell viability as well as colony-forming-unit percentage when compared to fresh lipoaspirate-derived SVF. On the other hand, the expression of stromal and adipogenic markers significantly enhanced in SVF following cryopreservation.

For clinical application it is important define xeno-free cryopreservation media as suggested by the FDA [52]. Lopez et al. [53] have evaluated two distinct cryopreservation xero-free media on the ASC post-thaw viability, plating efficiency rates, karyotype and differentiation potential. The results indicate that the medium composed of 3.5% DMSO, 3.5% ethylene glycol in DMEM/F-12 containing 0.5 M trehalose, 2% polyvinyl alcohol, 5% ficoll, 0.1 mM EGTA, 3 mM reduced glutathione and 5 mM ascorbic acid 2-phosphatase improves the plating efficiency rating to the level of the unfrozen cells also maintaining either multipotency or chromosomal normality.

To avoid FBS use, the addition to cryopreservation medium of different supplements such as human serum albumin (9%), human serum (90%) or knockout serum replacement (90%) to maintain the ASC recovery rate, viability, and growth kinetics at level similar to the conventional medium with 90% FBS, has been recently reported [29]. Gene expression, immunophenotype and multi-lineage differentiation ability of ASCs are not influenced by the different used cryopreservation media.

Cryopreserved cells can be subjected to freeze–thaw cycles. According to literature data, two rounds of ASC cryopreservation represent the maximum number [29,54]. In particular, when the cells were subjected to three or four freeze-thaw cycles, the viability, immunophenotype, gene expression and multi-lineage potential have been reported to be similar to fresh cells, even if a decreased replicative capacity with a longer doubling time has been reported [29].

## 7. Influence of Age and Obesity on ASC Isolation and Functions

Aging, obesity and related-chronic diseases can negatively affect ASCs and their microenvironment and impair their functions, thus leading to a low effectiveness of autologous cell therapy [55,56]. By contrast with bone-marrow-derived MSCs, the number of ASCs in adipose tissue does not decrease with age [57,58] even if their clonogenic and proliferative potential gradually declines [59,60,61,62,63]. ASCs isolated from old individuals have reduced function and adipogenic potential compared to ASCs from young subjects [64,65,66]. The growth rate of ASCs has been reported to be higher in younger (25–30 years old) than in older patients [61]. Also, paracrine activity of ASCs changes with age [67]. ASCs from obese subjects have been described with a reduced function and differentiation potential when compared to lean age-matched controls [66]. These changes should be considered for the definition of ASC-based therapy to verify the aging impact on the ASC properties (i.e., telomere lenght) [68], cell cycle inhibitor expression [69,70], proliferation [68,71], genomic integrity [72], reactive oxygen species (ROS) levels and antioxidant protective system [73].

In a recent review, Lowen et al. have collected the reports highlighting that ASCs in obesity are defective in various functionalities and properties including differentiation, angiogenesis, motility, multipotent state, metabolism and immunomodulation [74]. Deregulated ASCs, as well as other MSCs, have been suggested to play a crucial role in promoting the development of obesity [74,75]. Restoration of ASCs/MSCs might thus represent an additional strategy to combat obesity and its associated diseases. The undifferentiated multipotent state of ASCs is impaired in obese as compared to non-obese subjects. A reduced expression of multipotency-associated genes i.e., *OCT4*, *SAL4*, *SOX15* and *KLF4* and an upregulation of adipogenic and inflammatory genes are reported in obese ASCs [76,77]. In addition, ASCs isolated from obese adipose tissue show reduced telomerase activity and telomere length, indicative of a lower self-renewal capacity [78]. It has been reported that the body mass index (BMI) is associated to compromised osteogenesis potential of ASCs [79] and obese-derived ASCs differentiate much less toward osteogenic lineage than non-obese ASCs [80,81,82]. Impaired angiogenic potential is also shown in obese-derived ASCs [83]. In addition, obesity is also associated to an altered ASC secretome profile mainly due to the associated pro-inflammatory environment [76,84,85] able to negatively impact on the ASC differentiation potential and regenerative capability. Compared with lean-derived ASCs, ASCs derived from obese and T2DM subjects show reduced immunosuppressive activities and are less effective in suppressing lymphocyte proliferation and activating the M2 macrophage phenotype, thus contributing to obesity-associated inflammation and insulin resistance [86].

## Abbreviations

ASCs	Adipose-derived stem cells
MSCs	Mesenchymal stem cells
SVF	Stromal vascular fraction
MHC	Major histocompatibility complex
GMPs	Good manufacturing practices
IFATS	International Federation for Adipose Therapeutics and Science
ISCT	International Society for Cellular Therapy
MAL	Mechanical-Assisted Liposuction
PAL	Power-Assisted Liposuction
LAL	Laser-Assisted Liposuction
UAL	Ultrasound-Assisted Liposuction
CAL	Cell-Assisted Lipotransfer
FGF-basic	Fibroblast Growth Factor-basic
ROS	Reactive Oxygen Species

## References

[1] Bourin P., Bunnell B.A., Casteilla L., Dominici M., Katz A.J., March K.L., Redl H., Rubin J.P., Yoshimura K., Gimble J.M. (2013). Stromal cells from the adipose tissue-derived stromal vascular fraction and culture expanded adipose tissue-derived stromal/stem cells: A joint statement of the International Federation for Adipose Therapeutics and Science (IFATS) and the International Society for Cellular Therapy (ISCT). Cytotherapy.

[2] Dominici M., Le Blanc K., Mueller I., Slaper-Cortenbach I., Marini F.C., Krause D.S., Deans R.J., Keating A., Prockop D.J., Horwitz E.M. (2006). Minimal criteria for defining multipotent mesenchymal stromal cells. The International Society for Cellular Therapy position statement. Cytotherapy.

[3] Bajek A., Gurtowska N., Olkowska J., Maj M., Kazmierski L., Bodnar M., Marszalek A., Debski R., Drewa T. (2017). Does the Harvesting Technique Affect the Properties of Adipose-Derived Stem Cells? The Comparative Biological Characterization. J. Cell. Biochem..

[4] Hassan W.U., Greiser U., Wang W. (2014). Role of adipose-derived stem cells in wound healing. Wound Repair Regen..

[5] Raposio E., Bertozzi N. (2017). Isolation of Ready-to-Use Adipose-Derived Stem Cell (ASC) Pellet for Clinical Applications and a Comparative Overview of Alternate Methods for ASC Isolation. Curr. Protoc. Stem Cell Biol..

[6] Zuk P.A., Zhu M., Mizuno H., Huang J., Futrell J.W., Katz A.J., Benhaim P., Lorenz H.P., Hedrick M.H. (2001). Multilineage cells from human adipose tissue: Implications for cell-based therapies. Tissue Eng..

[7] Li S.H., Liao X., Zhou T.E., Xiao L.L., Chen Y.W., Wu F., Wang J.R., Cheng B., Song J.X., Liu H.W. (2017). Evaluation of 2 Purification Methods for Isolation of Human Adipose-Derived Stem Cells Based on Red Blood Cell Lysis With Ammonium Chloride and Hypotonic Sodium Chloride Solution. Ann. Plast. Surg..

[8] Palumbo P., Miconi G., Cinque B., La Torre C., Lombardi F., Zoccali G., Orsini G., Leocata P., Giuliani M., Cifone M.G. (2015). In Vitro Evaluation of Different Methods of Handling Human Liposuction Aspirate and Their Effect on Adipocytes and Adipose Derived Stem Cells. J. Cell. Physiol..

[9] Zuk P.A., Zhu M., Ashjian P., De Ugarte D.A., Huang J.I., Mizuno H., Alfonso Z.C., Fraser J.K., Benhaim P., Hedrick M.H. (2002). Human adipose tissue is a source of multipotent stem cells. Mol. Biol. Cell.

[10] Dubois S.G., Floyd E.Z., Zvonic S., Kilroy G., Wu X., Carling S., Halvorsen Y.D., Ravussin E., Gimble J.M. (2008). Isolation of human adipose-derived stem cells from biopsies and liposuction specimens. Methods Mol. Biol..

[11] Hendijani F. (2017). Explant culture: An advantageous method for isolation of mesenchymal stem cells from human tissues. Cell Prolif..

[12] Matsumoto D., Sato K., Gonda K., Takaki Y., Shigeura T., Sato T., Aiba-Kojima E., Iizuka F., Inoue K., Suga H. (2006). Cell-assisted lipotransfer: Supportive use of human adipose-derived cells for soft tissue augmentation with lipoinjection. Tissue Eng..

[13] Zielins E.R., Brett E.A., Longaker M.T., Wan D.C. (2016). Autologous Fat Grafting: The Science Behind the Surgery. Aesthet. Surg. J..

[14] Domenis R., Lazzaro L., Calabrese S., Mangoni D., Gallelli A., Bourkoula E., Manini I., Bergamin N., Toffoletto B., Beltrami C.A. (2015). Adipose tissue derived stem cells: In vitro and in vivo analysis of a standard and three commercially available cell-assisted lipotransfer techniques. Stem Cell Res. Ther..

[15] Banyard D.A., Salibian A.A., Widgerow A.D., Evans G.R.D. (2015). Implications for human adipose-derived stem cells in plastic surgery. J. Cell. Mol. Med..

[16] Francis M.P., Sachs P.C., Elmore L.W., Holt S.E. (2010). Isolating adipose-derived mesenchymal stem cells from lipoaspirate blood and saline fraction. Organogenesis.

[17] Shah F.S., Wu X.Y., Dietrich M., Rood J., Gimble J.M. (2013). A non-enzymatic method for isolating human adipose tissue-derived stromal stem cells. Cytotherapy.

[18] Yoshimura K., Shigeura T., Matsumoto D., Sato T., Takaki Y., Aiba-Kojima E., Sato K., Inoue K., Nagase T., Koshima I. (2006). Characterization of freshly isolated and cultured cells derived from the fatty and fluid portions of liposuction aspirates. J. Cell. Physiol..

[19] Zeng G.F., Lai K., Li J., Zou Y.Q., Huang H.L., Liang J., Tang X.D., Wei J., Zhang P.H. (2013). A rapid and efficient method for primary culture of human adipose-derived stem cells. Organogenesis.

[20] Busser H., De Bruyn C., Urbain F., Najar M., Pieters K., Raicevic G., Meuleman N., Bron D., Lagneaux L. (2014). Isolation of Adipose-Derived Stromal Cells Without Enzymatic Treatment: Expansion, Phenotypical, and Functional Characterization. Stem Cells Dev..

[21] Yoshimura K., Suga H., Eto H. (2009). Adipose-derived stem/progenitor cells: Roles in adipose tissue remodeling and potential use for soft tissue augmentation. Regen. Med..

[22] Coleman S.R. (2001). Structural fat grafts—The ideal filler?. Clin. Plast. Surg..

[23] Zhu M., Cohen S.R., Hicok K.C., Shanahan R.K., Strem B.M., Yu J.C., Arm D.M., Fraser J.K. (2013). Comparison of Three Different Fat Graft Preparation Methods: Gravity Separation, Centrifugation, and Simultaneous Washing with Filtration in a Closed System. Plast. Reconstr. Surg..

[24] Tremolada C., Colombo V., Ventura C. (2016). Adipose Tissue and Mesenchymal Stem Cells: State of the Art and Lipogems(R) Technology Development. Curr. Stem Cell Rep..

[25] Bellei B., Migliano E., Tedesco M., Caputo S., Picardo M. (2017). Maximizing non-enzymatic methods for harvesting adipose-derived stem from lipoaspirate: Technical considerations and clinical implications for regenerative surgery. Sci. Rep..

[26] Mizuno H., Tobita M., Uysal A.C. (2012). Concise Review: Adipose-Derived Stem Cells as a Novel Tool for Future Regenerative Medicine. Stem Cells.

[27] Agostini F., Rossi F.M., Aldinucci D., Battiston M., Lombardi E., Zanolin S., Massarut S., Parodi P.C., Da Ponte A., Tessitori G. (2018). Improved GMP compliant approach to manipulate lipoaspirates, to cryopreserve stromal vascular fraction, and to expand adipose stem cells in xeno-free media. Stem Cell Res. Ther..

[28] Devitt S.M., Carter C.M., Dierov R., Weiss S., Gersch R.P., Percec I. (2015). Successful Isolation of Viable Adipose-Derived Stem Cells from Human Adipose Tissue Subject to Long-Term Cryopreservation: Positive Implications for Adult Stem Cell-Based Therapeutics in Patients of Advanced Age. Stem Cells Int..

[29] Park S., Lee D.R., Nam J.S., Ahn C.W., Kim H. (2018). Fetal bovine serum-free cryopreservation methods for clinical banking of human adipose-derived stem cells. Cryobiology.

[30] Li J., Huang H.S., Xu X.M. (2017). Biological characteristics and karyotiping of a new isolation method for human adipose mesenchymal stem cells in vitro. Tissue Cell.

[31] Neri S., Bourin P., Peyrafitte J.A., Cattini L., Facchini A., Mariani E. (2013). Human Adipose Stromal Cells (ASC) for the Regeneration of Injured Cartilage Display Genetic Stability after In Vitro Culture Expansion. PLoS ONE.

[32] Vizoso F.J., Eiro N., Cid S., Schneider J., Perez-Fernandez R. (2017). Mesenchymal Stem Cell Secretome: Toward Cell-Free Therapeutic Strategies in Regenerative Medicine. Int. J. Mol. Sci..

[33] Makridakis M., Vlahou A. (2010). Secretome proteomics for discovery of cancer biomarkers. J. Proteom..

[34] Valadi H., Ekstrom K., Bossios A., Sjostrand M., Lee J.J., Lotvall J.O. (2007). Exosome-mediated transfer of mRNAs and microRNAs is a novel mechanism of genetic exchange between cells. Nat. Cell Biol..

[35] Chiellini C., Cochet O., Negroni L., Samson M., Poggi M., Ailhaud G., Alessi M.C., Dani C., Amri E.Z. (2008). Characterization of human mesenchymal stem cell secretome at early steps of adipocyte and osteoblast differentiation. BMC Mol. Biol..

[36] Kapur S.K., Katz A.J. (2013). Review of the adipose derived stem cell secretome. Biochimie.

[37] Kim H.S., Choi D.Y., Yun S.J., Choi S.M., Kang J.W., Jung J.W., Hwang D., Kim K.P., Kim D.W. (2012). Proteomic Analysis of Microvesicles Derived from Human Mesenchymal Stem Cells. J. Proteome Res..

[38] Varma M.J.O., Breuls R.G.M., Schouten T.E., Jurgens W.J.F.M., Bontkes H.J., Schuurhuis G.J., van Ham S.M., van Milligen F.J. (2007). Phenotypical and functional characterization of freshly isolated adipose tissue-derived stem cells. Stem Cells Dev..

[39] Bajek A., Gurtowska N., Gackowska L., Kubiszewska I., Bodnar M., Marszalek A., Januszewski R., Michalkiewicz J., Drewa T. (2015). Does the liposuction method influence the phenotypic characteristic of human adipose-derived stem cells?. Biosci. Rep..

[40] Almalki S.G., Agrawal D.K. (2016). Key transcription factors in the differentiation of mesenchymal stem cells. Differentiation.

[41] Maioli M., Basoli V., Santaniello S., Cruciani S., Delitala A.P., Pinna R., Milia E., Grillari-Voglauer R., Fontani V., Rinaldi S. (2016). Osteogenesis from Dental Pulp Derived Stem Cells: A Novel Conditioned Medium Including Melatonin within a Mixture of Hyaluronic, Butyric, and Retinoic Acids. Stem Cells Int..

[42] Basoli V., Santaniello S., Cruciani S., Ginesu G.C., Cossu M.L., Delitata A.P., Serra P.A., Ventura C., Maioli M. (2017). Melatonin and vitamin D interfere with the adipogenic fate of adipose-derived stem cells. Int. J. Mol. Sci..

[43] Johnstone B., Hering T.M., Caplan A.I., Goldberg V.M., Yoo J.U. (1998). In vitro chondrogenesis of bone marrow-derived mesenchymal progenitor cells. Exp. Cell Res..

[44] Choi Y.S., Dusting G.J., Stubbs S., Arunothayaraj S., Han X.L., Collas P., Morrison W.A., Dilley R.J. (2010). Differentiation of human adipose-derived stem cells into beating cardiomyocytes. J. Cell. Mol. Med..

[45] Fraser J.K., Schreiber R., Strem B., Zhu M., Alfonso Z., Wulur I., Hedrick M.H. (2006). Plasticity of human adipose stem cells toward endothelial cells and cardiomyocytes. Nat. Clin. Pract. Cardiovasc. Med..

[46] Fraser J.K., Wulur I., Alfonso Z., Hedrick M.H. (2006). Fat tissue: An underappreciated source of stem cells for biotechnology. Trends Biotechnol..

[47] Jang S., Cho H.H., Cho Y.B., Park J.S., Jeong H.S. (2010). Functional neural differentiation of human adipose tissue-derived stem cells using bFGF and forskolin. BMC Cell Biol..

[48] Banas A., Teratani T., Yamamoto Y., Tokuhara M., Takeshita F., Quinn G., Okochi H., Ochiya T. (2007). Adipose tissue-derived mesenchymal stem cells as a source of human hepatocytes. Hepatology.

[49] Charriere G., Cousin B., Arnaud E., Andre M., Bacou F., Penicaud L., Casteilla L. (2003). Preadipocyte conversion to macrophage—Evidence of plasticity. J. Biol. Chem..

[50] Urbich C., Dimmeler S. (2004). Endothelial progenitor cells—Characterization and role in vascular biology. Circ. Res..

[51] Zanata F., Bowles A., Frazier T., Curley J.L., Bunnell B.A., Wu X.Y., Wade J., Devireddy R., Gimble J.M., Ferreira L.M. (2018). Effect of Cryopreservation on Human Adipose Tissue and Isolated Stromal Vascular Fraction Cells: In Vitro and In Vivo Analyses. Plast. Reconstr. Surg..

[52] Halme D.G., Kessler D.A. (2006). FDA regulation of stem-cell-based therapies. N. Engl. J. Med..

[53] Lopez M., Bollag R.J., Yu J.C., Isales C.M., Eroglu A. (2016). Chemically Defined and Xeno-Free Cryopreservation of Human Adipose-Derived Stem Cells. PLoS ONE.

[54] Mamidi M.K., Nathan K.G., Singh G., Thrichelvam S.T., Yusof N.A.N.M., Fakharuzi N.A., Zakaria Z., Bhonde R., Das A.K., Sen Majumdar A. (2012). Comparative cellular and molecular analyses of pooled bone marrow multipotent mesenchymal stromal cells during continuous passaging and after successive cryopreservation. J. Cell. Biochem..

[55] Efimenko A.Y., Kochegura T.N., Akopyan Z.A., Parfyonova Y.V. (2015). Autologous Stem Cell Therapy: How Aging and Chronic Diseases Affect Stem and Progenitor Cells. Bior. Open Access.

[56] Palmer A.K., Tchkonia T., LeBrasseur N.K., Chini E.N., Xu M., Kirkland J.L. (2015). Cellular Senescence in Type 2 Diabetes: A Therapeutic Opportunity. Diabetes.

[57] De Girolamo L., Lopa S., Arrigoni E., Sartori M.F., Preis F.W.B., Brini A.T. (2009). Human adipose-derived stem cells isolated from young and elderly women: Their differentiation potential and scaffold interaction during in vitro osteoblastic differentiation. Cytotherapy.

[58] Harris L.J., Zhang P., Abdollahi H., Tarola N.A., DiMatteo C., McIlhenny S.E., Tulenko T.N., DiMuzio P.J. (2010). Availability of Adipose-Derived Stem Cells in Patients Undergoing Vascular Surgical Procedures. J. Surg. Res..

[59] Alt E.U., Senst C., Murthy S.N., Slakey D.P., Dupin C.L., Chaffin A.E., Kadowitz P.J., Izadpanah R. (2012). Aging alters tissue resident mesenchymal stem cell properties. Stem Cell Res..

[60] Huang S.C., Wu T.C., Yu H.C., Chen M.R., Liu C.M., Chiang W.S., Lin K.M. (2010). Mechanical strain modulates age-related changes in the proliferation and differentiation of mouse adipose-derived stromal cells. BMC Cell Biol..

[61] Schipper B.M., Marra K.G., Zhang W., Donnenberg A.D., Rubin J.P. (2008). Regional anatomic and age effects on cell function of human adipose-derived stem cells. Ann. Plast. Surg..

[62] Van Harmelen V., Skurk T., Rohrig K., Lee Y.M., Halbleib M., Aprath-Husmann I., Hauner H. (2003). Effect of BMI and age on adipose tissue cellularity and differentiation capacity in women. Int. J. Obes..

[63] Zhu M., Kohan E., Bradley J., Hedrick M., Benhaim P., Zuk P. (2009). The effect of age on osteogenic, adipogenic and proliferative potential of female adipose-derived stem cells. J. Tissue Eng. Regen. Med..

[64] Caso G., McNurlan M.A., Mileva I., Zemlyak A., Mynarcik D.C., Gelato M.C. (2013). Peripheral fat loss and decline in adipogenesis in older humans. Metabolism.

[65] Karagiannides I., Tchkonia T., Dobson D.E., Steppan C.M., Cummins P., Chan G., Salvatori K., Hadzopoulou-Cladaras M., Kirkland J.L. (2001). Altered expression of C/EBP family members results in decreased adipogenesis with aging. Am. J. Physiol. Regul. Integr. Comp. Physiol..

[66] Tchkonia T., Morbeck D.E., von Zglinicki T., van Deursen J., Lustgarten J., Scrable H., Khosla S., Jensen M.D., Kirkland J.L. (2010). Fat tissue, aging, and cellular senescence. Aging Cell.

[67] Siegel G., Kluba T., Hermanutz-Klein U., Bieback K., Northoff H., Schafer R. (2013). Phenotype, donor age and gender affect function of human bone marrow-derived mesenchymal stromal cells. BMC Med..

[68] Efimenko A.Y., Dzhoyashvili N.A., Kochegura T.N., Kalinina N.I., Akchurin R.S., Parfyonova Y.V. (2014). Adipose-Derived Stromal Cell (ADSC) for Autologous Cell Therapy: Disturbed Angiogenic Activity in Patients With Coronary Artery Disease and Diabetes Mellitus Type 2. Mol. Ther..

[69] Melzer D., Frayling T.M., Murray A., Hurst A.J., Harries L.W., Song H.L., Khaw K., Luben R., Surtees P.G., Bandinelli S.S. (2007). A common variant of the p16(INK4a) genetic region is associated with physical function in older people. Mech. Ageing Dev..

[70] Sethe S., Scutt A., Stolzing A. (2006). Aging of mesenchymal stem cells. Ageing Res. Rev..

[71] Efimenko A., Starostina E., Kalinina N., Stolzing A. (2011). Angiogenic properties of aged adipose derived mesenchymal stem cells after hypoxic conditioning. J. Transl. Med..

[72] Oh J., Lee Y.D., Wagers A.J. (2014). Stem cell aging: Mechanisms, regulators and therapeutic opportunities. Nat. Med..

[73] De Barros S., Dehez S., Arnaud E., Barreau C., Cazavet A., Perez G., Galinier A., Casteilla L., Planat-Benard V. (2013). Aging-related Decrease of Human ASC Angiogenic Potential Is Reversed by Hypoxia Preconditioning Through ROS Production. Mol. Ther..

[74] Louwen F., Ritter A., Kreis N.N., Yuan J. (2018). Insight into the development of obesity: Functional alterations of adipose-derived mesenchymal stem cells. Obes. Rev..

[75] Eljaafari A., Robert M., Chehimi M., Chanon S., Durand C., Vial G., Bendridi N., Madec A.M., Disse E., Laville M. (2015). Adipose Tissue-Derived Stem Cells From Obese Subjects Contribute to Inflammation and Reduced Insulin Response in Adipocytes Through Differential Regulation of the Th1/Th17 Balance and Monocyte Activation. Diabetes.

[76] Onate B., Vilahur G., Camino-Lopez S., Diez-Caballero A., Ballesta-Lopez C., Ybarra J., Moscatiello F., Herrero J., Badimon L. (2013). Stem cells isolated from adipose tissue of obese patients show changes in their transcriptomic profile that indicate loss in stemcellness and increased commitment to an adipocyte-like phenotype. BMC Genom..

[77] Patel R.S., Carter G., El Bassit G., Patel A.A., Cooper D.R., Murr M., Patel N.A. (2016). Adipose-derived stem cells from lean and obese humans show depot specific differences in their stem cell markers, exosome contents and senescence: Role of protein kinase C delta (PKCdelta) in adipose stem cell niche. Stem Cell Investig..

[78] Perez L.M., Bernal A., de Lucas B., San Martin N., Mastrangelo A., Garcia A., Barbas C., Galvez B.G. (2015). Altered Metabolic and Stemness Capacity of Adipose Tissue-Derived Stem Cells from Obese Mouse and Human. PLoS ONE.

[79] Frazier T.P., Gimble J.M., Devay J.W., Tucker H.A., Chiu E.S., Rowan B.G. (2013). Body mass index affects proliferation and osteogenic differentiation of human subcutaneous adipose tissue-derived stem cells. BMC Cell Biol..

[80] De Girolamo L., Stanco D., Salvatori L., Coroniti G., Arrigoni E., Silecchia G., Russo M.A., Niada S., Petrangeli E., Brini A.T. (2013). Stemness and Osteogenic and Adipogenic Potential Are Differently Impaired in Subcutaneous and Visceral Adipose Derived Stem Cells (Ascs) Isolated from Obese Donors. Int. J. Immunopathol. Pharmacol..

[81] Oliva-Olivera W., Gea A.L., Lhamyani S., Coin-Araguez L., Torres J.A., Bernal-Lopez M.R., Garcia-Luna P.P., Conde S.M., Fernandez-Veledo S., El Bekay R. (2015). Differences in the Osteogenic Differentiation Capacity of Omental Adipose-Derived Stem Cells in Obese Patients With and Without Metabolic Syndrome. Endocrinology.

[82] Strong A.L., Bowles A.C., Wise R.M., Morand J.P., Dutreil M.F., Gimble J.M., Bunnell B.A. (2016). Human Adipose Stromal/Stem Cells from Obese Donors Show Reduced Efficacy in Halting Disease Progression in the Experimental Autoimmune Encephalomyelitis Model of Multiple Sclerosis. Stem Cells.

[83] Onate B., Vilahur G., Ferrer-Lorente R., Ybarra J., Diez-Caballero A., Ballesta-Lopez C., Moscatiello F., Herrero J., Badimon L. (2012). The subcutaneous adipose tissue reservoir of functionally active stem cells is reduced in obese patients. FASEB J..

[84] Perez L.M., de Lucas B., Lunyak V.V., Galvez B.G. (2017). Adipose stem cells from obese patients show specific differences in the metabolic regulators vitamin D and Gas5. Mol. Genet. Metab. Rep..

[85] Silva K.R., Liechocki S., Carneiro J.R., Claudio-da-Silva C., Maya-Monteiro C.M., Borojevic R., Baptista L.S. (2015). Stromal-vascular fraction content and adipose stem cell behavior are altered in morbid obese and post bariatric surgery ex-obese women. Stem Cell Res. Ther..

[86] Serena C., Keiran N., Ceperuelo-Mallafre V., Ejarque M., Fradera R., Roche K., Nunez-Roa C., Vendrell J., Fernandez-Veledo S. (2016). Obesity and Type 2 Diabetes Alters the Immune Properties of Human Adipose Derived Stem Cells. Stem Cells.

